# The role of microRNAs in erectile dysfunction: From pathogenesis to therapeutic potential

**DOI:** 10.3389/fendo.2022.1034043

**Published:** 2022-10-25

**Authors:** Jingyu Song, Jiaxin Wang, Kang Liu, Wenchao Xu, Taotao Sun, Jihong Liu

**Affiliations:** ^1^ Department of Urology, Tongji Hospital, Tongji Medical College, Huazhong University of Science and Technology, Wuhan, China; ^2^ Institute of Urology, Tongji Hospital, Tongji Medical College, Huazhong University of Science and Technology, Wuhan, China

**Keywords:** erectile dysfunction, microRNA, expression, pathogenesis, therapy

## Abstract

Erectile dysfunction (ED) is a common male sexual dysfunction disease, and it was predicted that the number of ED patients worldwide will reach 322 million by 2025. However, the pathogenesis of ED is complex and the current treatment options are still limited, so it is urgent to explore new treatment strategies. Recent studies have shown that microRNAs (miRNAs) play an important role in ED, and these single-stranded non-coding small RNA molecules are involved in key pathophysiological processes in the occurrence and development of ED. Therefore, miRNAs have remarkable potential as therapeutic targets in ED. Here, this review introduces the physiological basis of erectile function and the pathophysiological changes in ED and summarizes the current knowledge on the expression, biological functions, and molecular mechanisms of miRNAs in ED, especially the potential of miRNA-targeted therapies to improve ED. This review will provide a comprehensive view of the role of miRNAs in the pathogenesis of ED and the potential value of miRNAs in the treatment of ED.

## Introduction

Erectile dysfunction (ED) is a term established by the 1993 NIH consensus conference on impotence, which refers to the persistent or recurrent inability to achieve or maintain sufficient penile erection to complete a satisfactory sexual life (1). As a common male sexual dysfunction disorder, ED has been shown to have a severe negative impact on an individual’s life, health, and well-being (2). Epidemiological investigation indicated that the prevalence of ED increases with age. It was reported that about 50% of men aged 40-70 years suffer from ED, while the prevalence of ED can rise to 70% in men over 70 years of age (3, 4).. In the past, ED was often considered to be mainly caused by psychological factors. However, current evidence showed that in addition to psychological factors, age, cardiovascular disease, diabetes, history of pelvic surgery, spinal cord injury, obesity, etc. were all crucial risk factors for ED (5–11). At present, the therapeutic methods of ED mainly included oral drugs, physical therapy, injection of active drugs in the cavernosal body, intraurethral alprostadil injection, and surgical treatment. Among them, oral phosphodiesterase type 5 inhibitor (PDE5i) is the most commonly used first-line treatment of ED in clinical practice (12). However, there are still a large number of ED patients with poor or no response to PDE5i. At the same time, the current therapeutic methods for ED cannot adequately meet the needs of the patients for sexual life (13). Therefore, the above problems prompt researchers to constantly explore and find novel methods for the treatment of ED.

**Figure 1:**
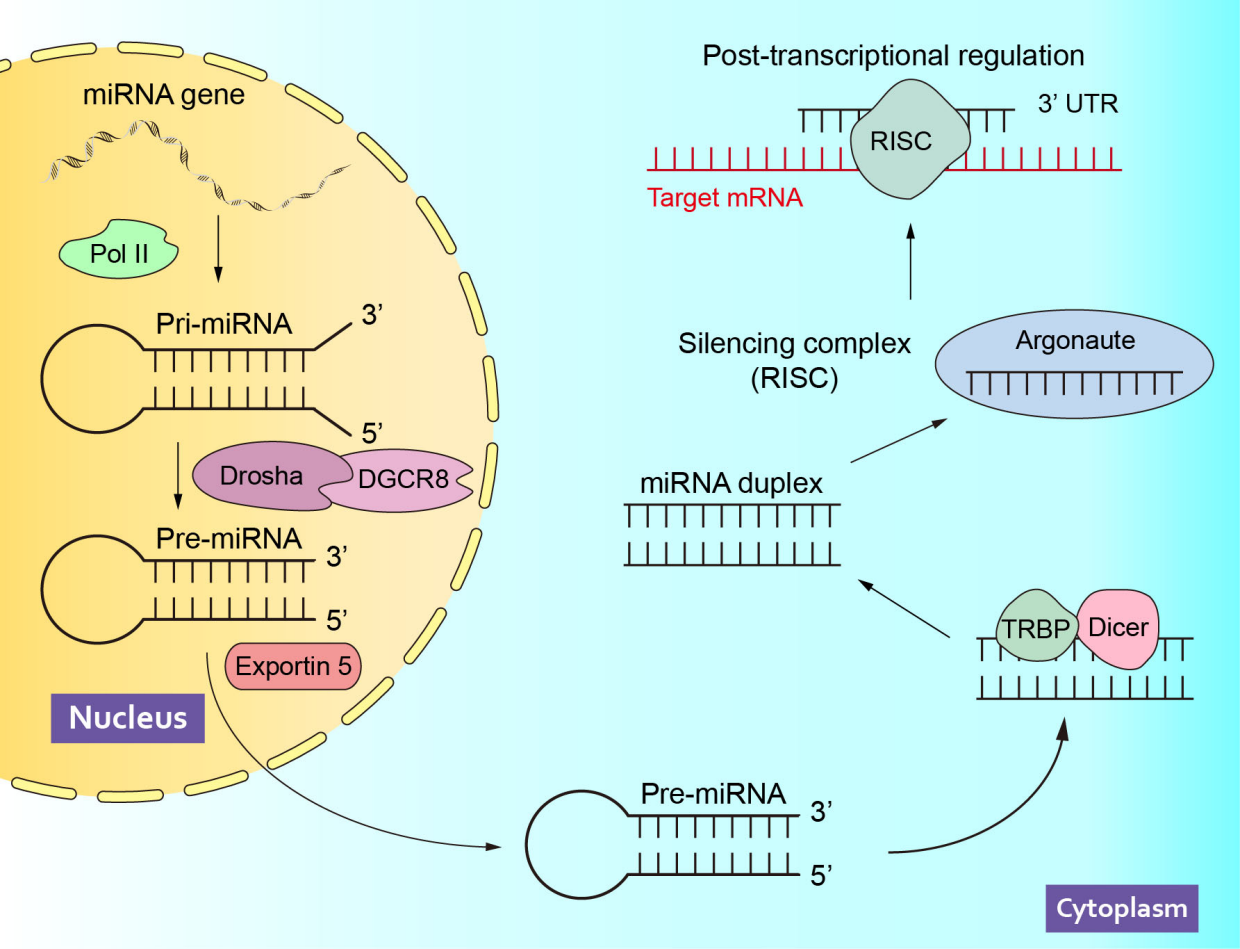
The Biogenesis and Function of miRNAs. Pol II, polymerase II; pri-miRNA, primary miRNAs; pre-miRNA, precursor miRNA; RISC, RNA-induced silencing complex; mRNA, messenger RNA; 3’ UTR, 3’-untranslated region.

MicroRNA (miRNA) is a single-stranded non-coding RNA molecule with a length of about 22 nucleotides (14, 15). Since lin-4 was reported as the first miRNA in 1993 (16), intensive research on miRNA has been conducted for more than two decades, which has led to a more detailed understanding of the epigenetic regulation process. In the nucleus, most miRNA genes are transcribed by RNA polymerase II (Pol II) to generate primary miRNAs (pri-miRNAs), which will be cleaved into precursor miRNA (pre-miRNA) by the Drosha, an endonuclease that binds to the double-stranded RNA-binding protein DGCR8/Pasha. Subsequently, pre-miRNAs are transported out of the nucleus and further cleaved into small double-stranded RNAs in the cytoplasm by Dicer, an endonuclease that binds to the double-stranded RNA-binding protein TRBP/Loquacious. Finally, one strand of the double-stranded RNA is loaded into the Argonaute protein to form the RNA-induced silencing complex (RISC), while the other strand is degraded (17). At present, miRNA has been verified to inhibit messenger RNA (mRNA) translation mainly by binding their complementary 3’-untranslated region (3’ UTR), thereby regulating gene expression at the post-transcriptional level (18, 19) (**Figure 1**). A series of studies have shown that miRNAs are involved in the occurrence and progression of various diseases, such as cardiovascular disease (20), diabetes (21), neurological dysfunction (22), metabolic syndrome (23) et al. Notably, miRNAs also play an important role in male sexual dysfunction (24) and reproductive dysfunction (25), and may serve as biomarkers and novel therapeutic targets for these diseases.

In recent years, great progress has been made in the treatment of ED worldwide. In addition to the improvement of existing treatment methods, stem cell therapy and gene therapy were considered to be promising forms of ED treatments (26, 27). As one of the key molecules for post-transcriptional regulation, miRNAs have been shown to serve as targets for stem cell and gene therapy for the treatment of ED (24, 28–30). However, the development of these therapeutic approaches still relies on in-depth studies on the pathogenesis of ED, continuous exploration of the miRNA mechanisms, and the identification of proper vectors for intervening miRNAs.

In this regard, this review will summarize the pathophysiology of ED, as well as a variety of miRNAs involved in ED-related pathophysiological processes, and discuss the potential roles of these miRNAs as biomarkers in the diagnosis and treatment of ED.

## The physiology of penile erection and the pathophysiology of ED

### The physiology of penile erection

**Figure 2:**
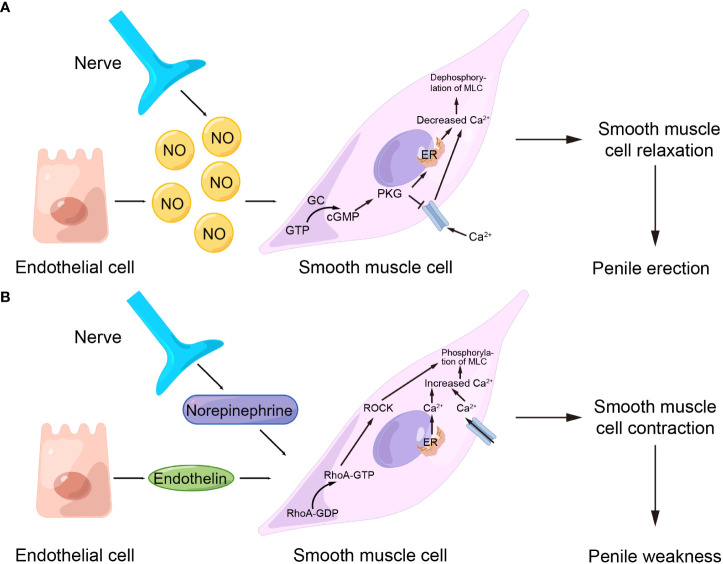
Important pathways that regulate penile contraction and relaxation. **(A)** NO/cGMP pathway. **(B)** RhoA/ROCK pathway. NO, nitric oxide; GTP, guanosine triphosphate; cGMP, cyclic guanylate phosphate; GC, guanylate cyclase; MLC, myosin light chain; ER, endoplasmic reticulum; ROCK, Rho-related protein kinase; GDP, guanosine diphosphate.

Normally, the contraction of the corpus cavernosum smooth muscle maintains the penis in a flaccid state. When sexual stimulation occurs, smooth muscle relaxation leads to local venous compression and blood reflux obstruction, resulting in erection (4). Therefore, the relaxation and contraction of corpus cavernosum smooth muscle are crucial for the regulation of penile erection. Current studies have indicated that multiple signaling pathways were involved in penile erection, among which the NO/cGMP pathway which mediates smooth muscle relaxation and the RhoA/ROCK pathway which mediates smooth muscle contraction were the most critical (12, 31, 32). In response to nitric oxide (NO), guanylate cyclase (GC) converts guanosine triphosphate (GTP) to cyclic guanylate phosphate (cGMP) and activates cGMP kinase in corpus cavernosum smooth muscle cells during its relaxation, which leads to the reduction of intracytoplasmic calcium ions, dephosphorylation of myosin light chain (MLC), and finally to smooth muscle relaxation and penile erection (33, 34) (**Figure 2A**). In contrast, in the process of smooth muscle contraction, under the action of substances such as norepinephrine and endothelin, the concentration of calcium ions in the cytoplasm of smooth muscle cells raises, and the GDP-binding Ras superfamily member RhoA is converted from RhoA-GDP to RhoA-GTP. Meanwhile, increased Ca^2+^ concentration also activates Rho-related protein kinase (ROCK), which expands the sensitivity of MLC to calcium ions, maintains MLC phosphorylation, and eventually promotes smooth muscle contraction and penile weakness (35–37) (**Figure 2B**).

### The pathophysiology of ED

Penile erection is a hemodynamic process accomplished by the corpus cavernosum under the joint action and mutual coordination of neurological, endocrine, and psychological factors, and is also influenced by multiple factors such as drugs, age, lifestyle habits, and systemic diseases (38). Abnormalities in one or more of these factors can lead to ED. ED can be divided into psychological ED, organic ED, or mixed psychological and organic ED, of which mixed ED is the most common (39).

Age is the most common risk factor in ED, the prevalence and the severity of ED increases with age, and age-related ED is one of the main types of ED being studied (40, 41). On the one hand, for men in the aging process, hypogonadism is one of the important reasons that may cause ED; on the other hand, the penis itself in the aging process of tissue and morphological changes also play a pivotal role in the occurrence of ED (41). Studies have shown that the aging of smooth muscle cells and endothelial cells in the penis can cause oxidative stress, which in turn causes an inflammatory reaction. Over time, the inflammation can cause cumulative damage to the penis, including disruption of vascular endothelial integrity and reduction in penile smooth muscle content (42, 43). It is also worth noting that most studies have demonstrated that aged-related ED has many commonalities in pathology with essential hypertension. Therefore, almost all risk factors that can cause primary hypertension can increase the incidence of ED, and likewise, ED can serve as an antecedent predictor of early cardiovascular events (44, 45).

Diabetes is the second most common risk factor for ED, with more than 50% of diabetics having ED as a complication and approximately 12% of patients with diabetes having ED as a first symptom (46, 47). As a complication of diabetes, the pathogenesis of diabetic ED is more complex and the extent of the disease is more severe (48). Studies have revealed that in the early stages of diabetes, vascular disorders and endothelial dysfunction occurred in the penis due to the effects of hyperglycemia, resulting in a decrease in NO production that would be produced by endothelial cells, as well as abnormal activation of the RhoA/ROCK pathway. These changes cause an imbalance in the diastolic and contractile functions of smooth muscle within the penis, causing diabetic ED. As the disease progresses, under the stimulation of long-term hyperglycemia and the accumulation of advanced glycation end products (AGEs), a large number of reactive oxygen species and pro-fibrotic factors were produced in the penile corpus cavernosum, which exacerbate smooth muscle apoptosis and eventually lead to structural changes such as fibrosis in the penis, thus further aggravating diabetes (49–52). In addition, diabetes caused by hypogonadism, neuropathy, and the patient’s psychological burden was also closely related to the development of ED (53–55).

Neurogenic ED is also the more commonly studied type of ED currently. Neurological ED is often associated with a variety of neurological disorders, such as multiple sclerosis, Parkinson’s disease, epilepsy, spinal cord injury, etc (56). Additionally, patients who have undergone radical pelvic surgery (e.g., radical cystectomy, radical prostatectomy) are at particularly high risk of neurogenic ED, mainly due to intraoperative damage to the cavernous nerves (57). Most studies have shown that in neurogenic ED, there was substantial apoptosis of smooth muscle and vascular endothelial cells in the penile corpus cavernosum with excessive collagen deposition, causing penile fibrosis (58–60).

As with neurogenic ED, research on obesity-related ED has increased in recent years. Because as known, obesity is a major global public health problem, and obesity is also associated with a high prevalence of ED. Studies have suggested that the main pathophysiological processes in obesity-related ED include oxidative stress (61, 62), inflammatory response (63, 64), and the resulting insulin (65, 66) and leptin resistance (67).

Epidemiological studies have revealed the common prevalence of psychological factors in the etiology of erectile dysfunction (4). In particular, depressive symptoms, pessimistic attitude, emotional stress, anxiety, etc. were closely related to psychogenic ED (68). Currently, it is believed that these negative emotional states may affect male sexual arousal through cognitive factors. Functional magnetic resonance imaging (fMRI) indicated that the medial preoptic area, amygdala, basal ganglia-thalamic-cortical circuit, and salience network were involved in chronic mild stress-mediated sexual arousal and erectile dysfunction in rats (69). In addition, dopamine D2 receptors in the basolateral amygdala and nucleus accumbens modulate erectile function in a rat model of chronic mild stress (70, 71). At the spinal level, acute severe stress significantly affected the spinal gastrin-releasing peptide (GRP) system, reducing GRP expression and androgen receptor (AR) response to circulating testosterone, resulting in suppressing the penile reflex (72). The dysfunction of the dopaminergic system is also an important cause of ED in depression model rats. The expression of dopamine receptor D2 and transporter solute carrier family 6 member 3 (SLC6A3) in the penis was decreased, and the catechol-O-methyltransferase (COMT) was increased in the depression model rats (73). Meanwhile, negative emotions led to the overactivation of adrenergic fibres, which release norepinephrine to act on α-receptors of the smooth muscle of the corpus cavernosum, activated RhoA/ROCK pathway to cause smooth muscle contraction, and inhibited penile erection (4).

Mixed ED is the most common etiological pattern in patients in clinical practice. In reality, there is a bidirectional relationship between organic ED and emotional disturbances, that is, organic ED itself can cause or aggravate depressive emotions, while moderate or severe depression can cause organic ED, which leads to the complexity of the pathophysiological process of mixed ED (74). In recent years, it has been found that the expression of endothelial nitric oxide synthase (eNOS) and neuronal nitric oxide synthase (nNOS) in the cavernosum of the chronic mild stress rat model and the levels of TNF-α, IL-1, and IL-6 in the corpus cavernosum of rats were increased, accompanied with decreased testosterone (75). Anyhow, compared with organic ED, the pathophysiology of psychogenic ED and mixed ED is still insufficient, and a large number of basic studies are needed to clarify the pathogenesis.

## miRNA expression in ED

This review provides a comprehensive overview of studies with the keywords (“microRNA” or “miRNA”) and (“erectile dysfunction”) published in the past 11 years (January 2011 to August 2022) using the Web of Science database in English. Fifty-six articles were identified based on the above search strategy. After excluding reviews and the articles not relevant to this review, a total of 44 articles were included in the subsequent review. Compared to other diseases, miRNA research in ED started relatively late, but the number of studies is increasing annually, indicating that miRNAs and their role in the development of ED are receiving more and more attention.

Most studies have now demonstrated that miRNA expression is abnormally up-or down-regulated in various diseases compared to normal conditions. Since a single miRNA can target hundreds of mRNAs, such altered miRNA expression is an influential factor affecting both disease initiation and progression (76, 77). With the development of bioinformatics and sequencing technologies, more and more aberrantly expressed miRNAs have been identified in various types of ED. Our review summarized these aberrantly expressed miRNAs in **Table 1**, which contained miRNAs that were up- and down-regulated in different ED types. These miRNAs have been shown in several studies to be involved in the development of ED with altered expression and may also serve as diagnostic markers for ED. **Table 2** summarizes the roles of some miRNAs in different ED.

**Table 1:** miRNAs were screened by microarray analysis or sequencing.

Author (Year, reference)	ED type	Specimen	Upregulated miRNA	Downregulated miRNA	Screening threshold
Pan et al. (78)	Age-related ED	Penis (rat)	miR-1, miR-29b, miR-183, miR-199a, miR-200a, miR-200b, miR-200c, miR-203, miR-205, miR-206	miR-98, miR-125a, miR-127, miR-133a, miR-148, miR-196b, miR-322, miR-324, miR-328, miR-351, miR-379, miR-434, miR-494, miR-532, miR-541, miR-674	Fold change > 2
Pan et al. (79)	Diabetic ED	Penis (mouse)	miR-18a, miR-190a, miR-206, miR-210, miR-215, miR-290b, miR-542	miR-10a, miR-122, miR-133a, miR-224, miR-299b, miR-301a, miR-338, miR-379, miR-410, miR-451b, miR-466, miR-485, miR-96, miR-99a	Fold change ≥ 3
Kang et al. (80)	Diabetic ED	Penis (rat)	miR-1298, miR-337-3p, miR-134-5p, miR-503-5p, miR-148a-5p	miR-202-5p, miR-122-5p, miR-1b, miR-204-5p, miR-145-3p, miR-6321, miR-743a-3p, miR-463-5p, miR-211-5p, miR-741-3p, miR-881-3p, miR-743b-5p, miR-871-5p, miR-743b-3p, miR-471-5p	Fold change > 2
Liu et al. (81)	Neurogenic ED	Penis (rat)	miR-451, miR-144, miR-3065, miR-338, miR-872, miR-138-2, miR-142, miR-324, miR-425, miR-181b-1, let-7i, miR-103-1, miR-93, miR-101a, miR-101b, miR-21, miR-674, miR-10a, miR-139, miR-138-1, miR-29b-2	–	Fold change > 2
Barbery et al. (82)	Obesity-related ED	Penis (mouse)	miR-151-5p, miR-1937c, miR-720, miR-1937a, miR-205	miR-550, miR425, miR-134, miR-153, and miR-26b and 60 other miRNAs (no detail mentioned)	Fold change > 2
Bai et al. (83)	Obesity-related ED	Penis (rat)	miR-200a-3p, miR-370-3p, miR-26b-5p, miR-215, miR-134-5p, miR-10b-5p, miR-142-3p, miR-96-5p, miR-541-5p, miR-92a-3p, miR-150-5p, miR-206-3p, miR-210-3p, miR-340-3p, miR-218a-5p, miR-376b-3p, miR-342-3p, miR-203a-3p, miR-194-5p, miR-22-5p, miR-23a-3p, miR-101b-3p, miR-298-5p, miR-33-5p, miR-301a-3p, miR-450a-5p, miR-540-3p, miR-128-3p, miR-137-3p, miR-183-5p, miR-1-5p, miR-328a-3p, miR-100-5p, miR-25-3p, miR-122-5p, miR-103-3p, miR-323-3p, miR-30a-5p, let-7d-3p, miR-365-3p, miR-19a-3p, miR-539-5p, miR-297, miR-487b-3p, miR-32-5p, miR-292-3p, miR-349, miR-30a-3p	miR-127-3p, miR-223-3p, miR-409a-5p, miR-142-5p, miR-124-3p, miR-99b-5p, miR-207, miR-382-5p, miR-24-1-5p, miR-34b-5p, let-7b-5p, miR-361-5p, miR-341, miR-483-3p, miR-18a-5p, let-7a-5p, miR-140-3p, miR-489-3p, miR-499-5p, miR-187-3p	Fold change≥1.5

ED, erectile dysfunction.

**Table 2:** The expression and function of differentially expressed miRNAs in various EDs.

ED Types	miRNA	Specimen	Differential Expression	Identification method	Function	Reference
Age-related ED	miR−200a	penis (rat)	up	Microarray qRT-PCR	attenuate endothelial function via SIRT1 inhibition	(78, 84)
	miR-1	penis (rat)	up	Microarray qRT-PCR	contribute to apoptosis, fibrosis, and endothelium dysfunction (bioinformatic analysis)	(78)
	miR-203	penis (rat)	up	Microarray qRT-PCR	contribute to apoptosis, fibrosis, and endothelium dysfunction (bioinformatic analysis)	(78)
	miR-206	penis (rat)	up	Microarray qRT-PCR	contribute to apoptosis, fibrosis, and endothelium dysfunction (bioinformatic analysis)	(78)
Diabetic ED	miR-874-3p	penis (rat)	down	qRT-PCR	binds to Nupr1 and inhibits Nupr1/Chop-mediated pathway	(85)
	miR-205	penis (rat)	up	qRT‐PCR	contribute to the pathogenesis of diabetic ED via down‐regulation of androgen receptor expressions	(73)
	miR-21-5p	penis (rat)	down	qRT‐PCR	suppressed PDCD4 expression and ED in rats with DM	(86)
	miR-93	blood (human)	up	qRT‐PCR	prospective markers	(87)
	miR-320	blood (human)	up	qRT‐PCR	prospective markers	(87)
	miR-16	blood (human)	up	qRT‐PCR	prospective markers	(87)
	miR-141	penis (rat)	down	qRT‐PCR	miR‐141 binds to NGFRAP1 and restores the erectile function via downregulation of NGF/p75NTR signaling	(88)
	miR-18a	penis (mouse)	up	Microarray qRT-PCR	contribute to apoptosis, fibrosis, eNOS/cGMP/PKG, and vascular smooth muscle contraction processes (bioinformatic analysis)	(79)
	miR-206	penis (mouse)	up	Microarray qRT-PCR	contribute to apoptosis, fibrosis, eNOS/cGMP/PKG, and vascular smooth muscle contraction processes (bioinformatic analysis)	(79)
	miR-122	penis (mouse)	down	Microarray qRT-PCR	contribute to apoptosis, fibrosis, eNOS/cGMP/PKG, and vascular smooth muscle contraction processes (bioinformatic analysis)	(79)
	miR-133	penis (mouse)	down	Microarray qRT-PCR	contribute to apoptosis, fibrosis, eNOS/cGMP/PKG, and vascular smooth muscle contraction processes (bioinformatic analysis)	(79)
	miR-6321	penis (rat)	up	miRNA Sequencing	contribute to cellular response to growth factor stimulus angiogenesis, positive regulation of apoptotic process (bioinformatic analysis)	(80)
	miR-122-5p	penis (rat)	up	miRNA Sequencing	contribute to cellular response to growth factor stimulus angiogenesis, positive regulation of apoptotic process (bioinformatic analysis)	(80)
	miR-1298	penis (rat)	down	miRNA Sequencing	miR-1298/B4GalT1 axis might exert function in stem cell therapy for ED	(80)
Neurogenic ED	miR-33	penis (rat)	up	qRT-PCR	preserved the erectile function of BCNI rats through regulating miR-33/GDNF axis	(89)
	miR-101a	penis (rat)	up	miRNA Sequencing qRT-PCR	regulate the processes of cell proliferation, differentiation, apoptosis, inflammation, and fibrosis (bioinformatic analysis)	(81)
	miR-138	penis (rat)	up	miRNA Sequencing qRT-PCR	regulate the processes of cell proliferation, differentiation, apoptosis, inflammation, and fibrosis (bioinformatic analysis)	(81)
	miR-338	penis (rat)	up	miRNA Sequencing qRT-PCR	regulate the processes of cell proliferation, differentiation, apoptosis, inflammation, and fibrosis (bioinformatic analysis)	(81)
	miR-142	penis (rat)	up	miRNA Sequencing qRT-PCR	regulate the processes of cell proliferation, differentiation, apoptosis, inflammation, and fibrosis (bioinformatic analysis)	(81)
Obesity-related ED	miR-720	penis (mouse)	up	Microarray	no validation	(82)
	miR-1937a	penis (mouse)	up	Microarray	no validation	(82)
	miR-1937c	penis (mouse)	up	Microarray qRT-PCR	no validation	(82)
	miR-205	penis (mouse)	up	Microarray	no validation	(82)
	miR-151-5p	penis (mouse)	up	Microarray qRT-PCR	no validation	(82)
	miR-550	penis (mouse)	down	Microarray	no validation	(82)
	miR-425	penis (mouse)	down	Microarray qRT-PCR	no validation	(82)
	miR-134	penis (mouse)	down	Microarray	no validation	(82)
	miR-153	penis (mouse)	down	Microarray qRT-PCR	no validation	(82)
	miR-26b	penis (mouse)	down	Microarray	no validation	(82)
	miR-328a	penis (rat)	up	Microarray qRT-PCR	decrease the signaling mediator HMOX1/HO-1 of erectile function expression	(83)

ED, erectile dysfunction; qRT-PCR, quantitative real-time polymerase chain reaction.

As shown in **Table 1**, in aged-related ED, Pan et al. identified four upregulated miRNAs (miR-200a, miR-1, miR-203, and miR-206) by microarray analysis of miRNA expression and validation by quantitative real-time polymerase chain reaction (qRT-PCR) techniques on penile tissue from 18-month-old ED rats and 3-month-old young rats (78, 84). All of the abnormally expressed miRNAs were associated with endothelial dysfunction and were essential factors affecting erectile function in aged rats.

In diabetic ED, related studies from multiple research groups validated a total of six up-regulated miRNAs and five down-regulated miRNAs. Specifically, among the upregulated miRNAs, Jiang et al. found upregulated expression of miR-93, miR-320, and miR-16 in the serum of patients suffering from diabetic ED by qRT-PCR, suggesting that these miRNAs might be useful for the early diagnosis of diabetic ED (87). Using a rat model of diabetic ED, Wen et al. demonstrated that miR-205 expression was upregulated in penile tissue by qRT-PCR and could inhibit the androgen receptor (AR) (90). In addition, Pan et al. screened 21 differentially expressed miRNAs (fold change ≥ 3) in penile tissues of diabetic rats by GeneChip array techniques (Affymetrix miRNA 4.0 Array) and applied qRT-PCR to confirm that miR-18a and miR-206 were upregulated in penile tissues and inhibited insulin-like growth factor (IGF-1), while miR-122 and miR-133 were downregulated in penile tissues of diabetic ED rats (79). Besides, among other down-regulated miRNAs, Wen et al. verified that miR-141 was down-regulated in penile tissues of rats with diabetic ED by qRT-PCR and demonstrated that miR-141 had a protective effect on the smooth muscle in the corpus cavernosum (88). Also, miR-141 was proved to have a suppressive effect on the RhoA/ROCK pathway and thus decreased miR-141 expression was associated with the development of diabetic ED (91). Huo et al. comfirmed that miR-874-3p and miR-21-5p expression were downregulated in diabetic ED rats by qRT-PCR and correlated with apoptosis of smooth muscle cells (85, 86). Currently, Kang et al. identified that miR-6321 and miR-122-5p were up-regulated, while miR-1298 was down-regulated in diabetic ED rats with miRNA sequencing (80).

In neurogenic ED, Liu et al. identified 124 aberrantly expressed miRNAs using RNA-seq in a rat model of bilateral cavernous nerve injury and subsequently verified four of them, miR-101a, miR-138, miR-338, and miR-142, as up-regulated miRNAs by qRT-PCR (81). Zheng et al. also reported the upregulation of miR-33 using the same model and further investigated that the upregulation of miR-33 suppressed neurotrophic factors involved in the ED recovery process (89).

In obesity-related ED, Barbery et al. applied the quantifiable miRNA profiling technique (NanoString) to the penile corpus cavernosum of mice on a high-fat diet (HFD) and identified 5 up-regulated miRNAs and 65 down-regulated miRNAs (fold change ≥ 2). However, in the qRT-PCR validation, they found only miR-1937c and miR-151-5p significantly upregulated in HFD mice, whereas the other three (miR-720, miR-1937a, miR-205) identified by microarray trended in the correct direction. Among the down-regulated miRNAs, they only verified the statistically different expression of miR-153 and miR-425 by qRT-PCR, and a large number of down-regulated miRNAs screened by microarray were not verified (82). Notably, as described above, miR-205 has also been reported to be upregulated in diabetic ED (90). Bai et al. examined the miRNA expression profile of obese rat spongy tissue by miRNA microarray analysis and found 68 differentially expressed miRNAs (fold change ≥ 1.5), and applied PCR to verify that miR-328a were upregulated in obese rats (83).

## Biological functions and molecular mechanisms of miRNA in ED

### Apoptosis

Apoptosis is an important biological process in the development of ED. Under various stimuli such as high glucose, reactive oxygen species, and inflammation, smooth muscle cells or endothelial cells in the penile corpus cavernosum undergo apoptosis, which affects erection (92, 93). Multiple studies have now suggested that miRNAs play a bidirectional role in the modulation of apoptosis in ED. On the one hand, miRNAs can promote apoptosis in some pathological situations. For example, Wen et al. demonstrated that miR-205 can inhibit the androgen receptor, thus causing smooth muscle apoptosis in the penile corpus cavernosum of diabetic ED rats (90). Another example is that Liu et al. predicted that four miRNAs, miR-101a, miR-138, miR-338, and miR-142, might be involved in apoptosis in neurogenic ED by gene ontology analysis and the Kyoto Encyclopedia of Genes and Genomes pathway analysis of the target genes (81). Among them, miR-101a and miR-338 can directly regulate genes related to vascular smooth muscle contraction, while miR-142 and miR-138 can regulate apoptosis through calcium signaling pathway. Recently, Zhou et al. applied comprehensive transcriptome analysis to identify that miR-484-x and miR-653-5p were regulated by lncRNA ENSRNOT00000029245 to promote apoptosis of cavernosal smooth muscle, endothelial cells and ED in geriatric rats (94). On the other hand, miRNAs can also inhibit apoptosis by targeting the expression of specific genes to inhibit apoptosis, thus providing an ameliorative effect on ED. For instance, the Nupr1/Chop pathway is an endoplasmic reticulum stress pathway that mediates apoptosis. Huo et al. found that miR-874-3p can inhibit Nupr1 and thereby downregulate the Nupr1/Chop pathway in diabetic ED, exerting an anti-apoptotic effect (85, 95). NGFRAP1 is a nerve growth factor receptor-associated protein that interacts with the neurotrophic factor receptor p75 and mediates apoptosis. Wen et al. found that miR-141 inhibited NGFRAP1 in diabetic ED, resulting in reduced apoptosis (88). PDCD4 has various functions such as cell growth inhibition and apoptosis induction and is an essential regulator mediating apoptosis in vascular smooth muscle cells (96). Huo et al. found that miR-21-5p could inhibit the expression of PDCD4 in diabetic ED rats and reduce smooth muscle apoptosis (86).

### Fibrosis

In ED, fibrosis is an important pathological change in the penile corpus cavernosum, which is mainly characterized by the massive proliferation of pro-fibrotic factors and deposition of collagen (97). It has been reported that miR-101a, miR-138, miR-338 and miR-142 may be involved in ED fibrosis through TGF-β signaling pathway and Wnt signaling pathway (81). In contrast, miR-145, miR-let7b, and miR-let7c were validated to play an anti-fibrotic role in ED (98, 99). miR-145 has been associated with the TGF-β receptor subsequent pathways. As an important pro-fibrotic cytokine, TGF-β has been described in detail in numerous studies as a major factor involved in penile corpus cavernosum fibrosis, and which induces fibrosis through both SMAD and non-SMAD signaling pathways (100). Liu et al. revealed that miR-145 inhibits the TGF-β receptor, which in turn reduced collagen deposition by inhibiting the SMAD2 signaling pathway (99). In another study, Zhu et al. found that miR-let7b and miR-let7c in exosomes derived from adipose stem cells could play an anti-apoptotic role in diabetic ED and the exosomes encapsulating miR-let7b and miR-let7c were critical for reducing collagen deposition in penile tissue (98).

### Angiogenesis

In recent years, there has been a new understanding of the role of the vascular endothelium in erection and the impact of vascular dysfunction on ED. Damage to the vascular endothelium can lead to vascular dysfunction, which in turn can cause ED. In contrast, the use of vascular growth factors to promote revascularization can effectively improve ED (101, 102). RNA sequencing data from Ouyang et al. showed that miR-21-5p, let-7 family, miR-10 family, miR-30 family, and miR-148a-3p from human urinary-derived stem cell exosomes can promote angiogenesis in diabetic ED (103). Meanwhile, miR-126, miR-130a, and miR-132 from adipose stem cell exosomes were also revealed to promote angiogenesis in diabetic ED (98). In a recent study, Zou et al. also investigated the pro-angiogenic effects of miR-126 in the ED, showing that miR-126 regulated a variety of transcription factors that regulate cell growth, such as IRS1 and KLF10, thereby promoting angiogenesis and improving erectile function (104). Alternatively, vascular endothelial growth factor (VEGF) is a well-known pro-angiogenic substance that stimulates the growth and proliferation of endothelial cells and has an important regulatory role in angiogenesis (105). Wang et al. proposed in a rat model of neurogenic ED that miR-200a inhibits VEGF and is an essential target in the development of ED (106).

### NO/cGMP pathway

The NO/cGMP pathway is currently one of the most well-studied and important pathways in ED. As described in the previous section on erectile physiology, normal levels of NO/cGMP pathway-related molecules directly affect erectile function. Currently, a variety of miRNAs reported in relevant studies all negatively regulated the NO/cGMP pathway, including miR-328 (102, 107), miR-200a (78, 84), miR-1 (78), miR-203 (78), miR-206 (78, 79), miR-18a (79), miR-155 (108), and miR-146a (109). In particular, miR-328 has been shown to inhibit the NO/cGMP pathway by studies from different groups. Bai et al. suggested that miR-328 could reduce cGMP levels by inhibiting HO-1 in an obese ED rat model (83), while Li et al. found that the miRNA could directly target and inhibit eNOS by using a diabetic ED rat model (107). Pan et al. first found that miR-200a, miR-1, miR-203, and miR-206 could regulate the NO/cGMP pathway in senile ED by bioinformatics analysis (78), and then further demonstrated that miR-200a could inhibit SIRT1, which in turn affected eNOS and cGMP and reduced endothelial function (84). In another study by pan et al., they also elaborated on the possible regulatory role of miR-206 as well as miR-18a on the NO/cGMP pathway in diabetic ED (79). In addition, Rocha et al. showed that miR-155 inhibits eNOS, and that exercise, diet control, and atorvastatin treatment can reduce miR-155 expression, thereby improving endothelial dysfunction (108). Ding et al. found that miR-146a could directly target nNOS inhibition, while inhibition of miR-146a significantly increased the level of nNOS (109).

### Other biological functions and molecular mechanisms

**Figure 3:**
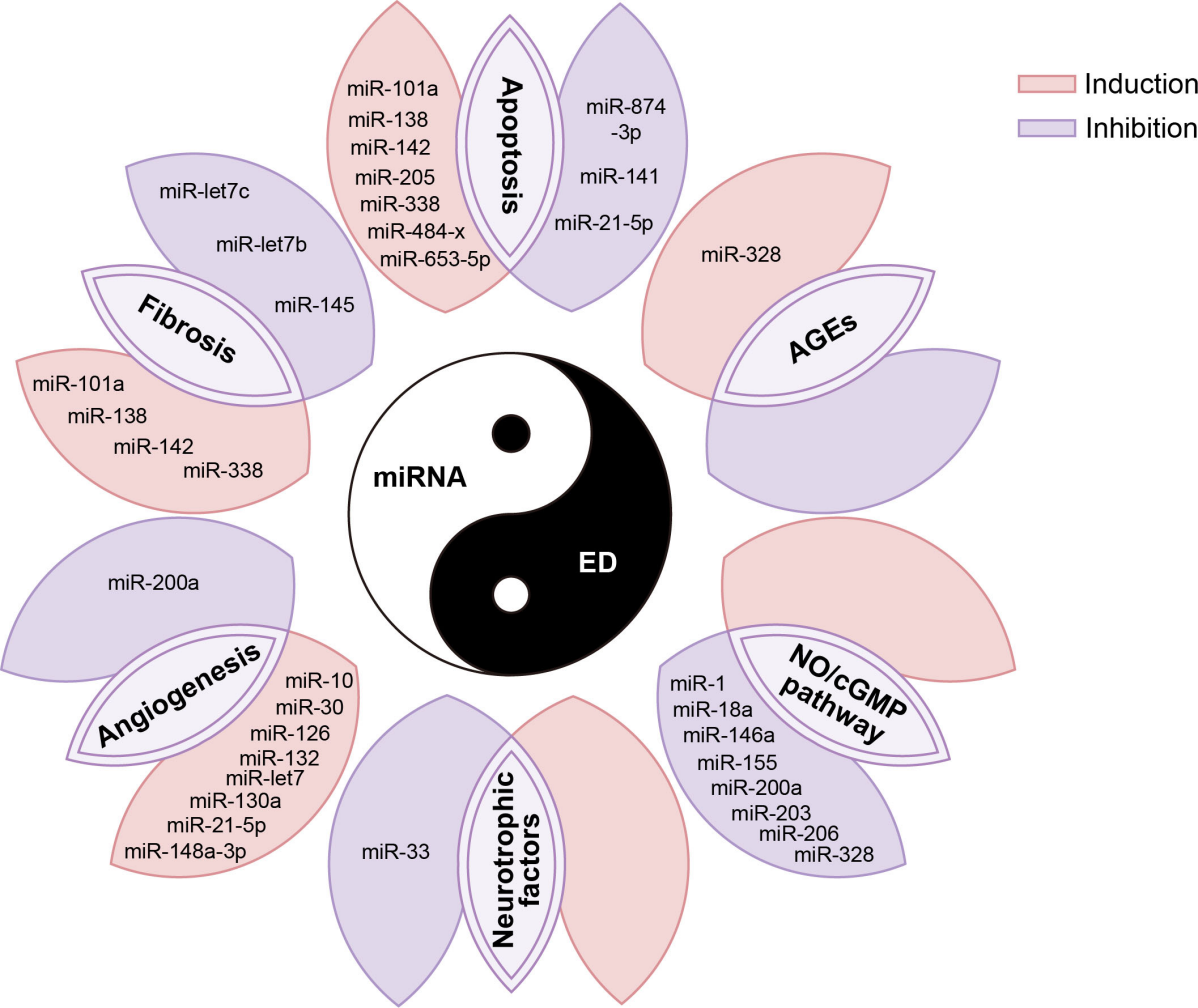
miRNAs are involved in various pathophysiological processes in the occurrence and development of ED. AGE, advanced glycation end products; NO, nitric oxide; cGMP, cyclic guanylate phosphate.

In addition, miRNAs also have regulatory effects on AGEs, neurotrophic factors, and other biological factors involved in the pathophysiology of ED. For example, studies have reported that inhibition of miR-328 reduces AGEs in diabetic ED (107), while miR-33 inhibits GDNF, a neurotrophic factor involved in the neurological ED response process (89). However, at present, the number of reports on the regulatory effects of miRNA on these biological factors in ED is relatively less, which needs to be further confirmed by more studies in the future. We summarized the biological functions and molecular mechanisms of these above miRNAs in the ED in **Figure 3**.

## Therapeutic potentials of miRNA in ED

miRNAs play an influential role in all stages of ED and thus can be used as potential targets for ED therapeutic interventions, which opens a new window for the development of ED therapeutics. Here, this review will discuss various miRNA-based therapeutic strategies for ED based on the available relevant literature.

**Figure 4:**
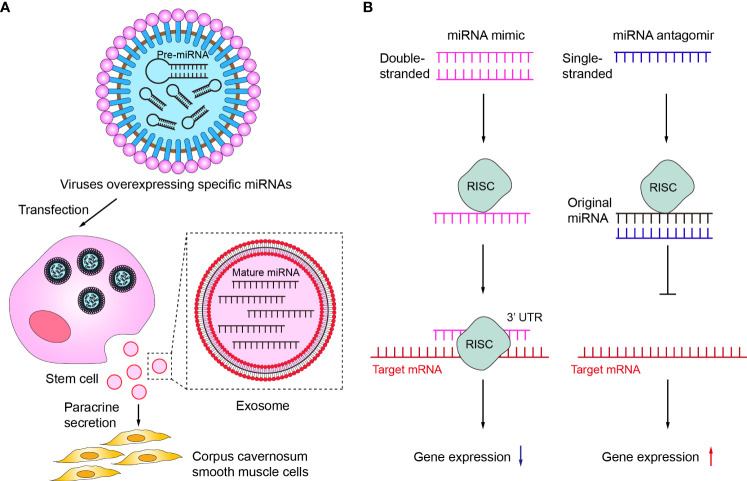
Therapeutic potentials of miRNA in ED. **(A)** Delivery of corresponding miRNAs *via* stem cells or exosomes. **(B)** The mechanisms of miRNA mimic and miRNA antagomir. RISC, RNA-induced silencing complex.

First, a variety of miRNAs have been summarized and described in detail in the above review as having protective effects on erectile function and improving effects on ED. However, the expression of some of these miRNAs was usually down-regulated in ED, so targeted supplementation of these under-represented miRNAs may be an effective ED treatment option. Currently, the more studied miRNA supplementation methods in the ED mainly included the delivery of corresponding miRNAs *via* stem cells or exosomes (**Figure 4A**). Among them, the main idea of using stem cells to deliver miRNA is to first transfect viruses overexpressing specific miRNAs into stem cells and subsequently transplant the stem cells into the penile corpus cavernosum to increase the local content of specific miRNAs in the penis through paracrine secretion of stem cells. For example, Zou et al. transplanted miR-126 over-expressing lentivirus muscle stem cells into the penile corpus cavernosum of ED rats and found that the erectile function of the rats was significantly improved after transplantation (104). Alternatively, an increasing number of studies in recent years have shown that increasing miRNA content through direct injection of stem cell-secreted exosomes can also be effective in improving ED (86, 98, 103, 110). As non-viral and non-cellular substances, exosomes have the advantages of high safety and low immunogenicity, and can directly deliver the carried miRNAs into cells. Therefore, exosomes may be the main carriers for delivering miRNAs in the future (111, 112).

Secondly, miRNA mimic or miRNA agomir is also a common current regulatory tool to target insufficient levels of specific miRNAs (**Figure 4B**). miRNA mimic is a chemically synthesized double-stranded RNA molecule that mimics the function of an endogenous miRNA, which can mimic miRNAs that are downregulated due to disease (113). Wen et al. demonstrated that miR-141 mimic can effectively compensate for the deficiency of miR-141 in diabetic ED rats, increase the pressure in the penile corpus cavernosum of rats and improve erectile function (88). miRNA agomir is a special chemical modified miRNA agonist that acts similarly to the miRNA mimic. Experimental animal studies by Huo et al. have demonstrated that supplementation with miR-874-3p agomir can effectively improve ED (85). In contrast to miRNA mimic or miRNA agomir, miRNA inhibitor or miRNA antagomir can selectively inhibit miRNA (114, 115). Currently, both miRNA inhibitor and miRNA antagomir have been applied in ED therapeutic studies to reduce miRNAs that are abnormally highly expressed in the ED to delay or prevent the progression of ED. Wen et al. reported that the miR-205 inhibitor reduced the inhibitory effect of miR-205 on androgen receptors in diabetic ED rats to improve erectile function (90). Li et al. used miR-328 antagomir to antagonize the inhibitory effect of miR-328 on eNOS in diabetic ED rats, thereby increasing the content of cGMP in rat penile tissue (107).

In addition, there are biomolecules or related drugs that target miRNAs that have the potential to be novel approaches for the treatment of ED. Recent studies have shown that long non-coding RNAs can act as competitive endogenous RNA that binds to miRNA interactions and acts as an inhibitor of miRNA (116, 117). Wang et al. used lncRNA-MIAT, which reduced the ability of miR-200a to interfere with mRNA-encoded proteins of target genes through its targeted competitive adsorption on miR-200a, thus promoting the differentiation of bone marrow stem cells to endothelial cells and acting as a therapeutic agent for ED (106). Icariin II is a flavonoid isolated from the traditional Chinese medicine Epimedium, which has been shown to have a therapeutic effect on ED (118). In recent studies, Icariin II has been shown to inhibit specific miRNAs in neurogenic ED, thereby promoting the differentiation of adipose stem cells to Schwann cells to improve neurogenic ED (89, 119).

## Discussion

In recent years, studies on the involvement of miRNAs in disease development, diagnosis, and treatment were growing rapidly, and the understanding of the potential role of miRNAs in ED had increased significantly. miRNA aberrant expression was closely associated with ED, and miRNAs played an important role in key biological processes such as penile corpus cavernosum fibrosis, apoptosis, angiogenesis, and NO release, which highlighted its potential as a therapeutic target in ED. Increasing down-regulated miRNAs that promote erectile function and decreasing up-regulated miRNAs that inhibit erectile function through various approaches have great value and promise for the development of new ED therapeutic agents.

Despite the vigorous development of miRNA-related studies in ED, there are still some limitations in existing studies. We are delighted to see that we can screen out a large number of miRNAs by microarray and miRNA sequencing that may play an important role in the pathogenesis of ED. Some molecules such as miR-200a and miR-122 are functional in the ED by different research groups. However, the vast majority of molecules screened by microarray or sequencing were not validated by qRT-PCR, making them less credible. In addition, the fold change for identifying differentially expressed miRNAs varied between studies, making it difficult to compare these studies. Sequencing studies of human blood samples to provide a comprehensive view of miRNAs in ED patients are still lacking. It should not be ignored that the proportion of psychogenic ED and mixed ED in clinical patients is gradually increasing, but so far, there is no relevant report on miRNA and non-organic ED.

In the therapeutic field, although a large number of animal experiments involving miRNAs for the treatment of ED have been conducted in recent years, none of the miRNAs have entered clinical trials so far. One of the biggest challenges in developing miRNA-based therapies is identifying the best miRNA targets for each ED type. However, existing basic research is sketchy in screening miRNA targets and lacks strong experimental validation. miRNA is a double-edged sword. On the one hand, it can act on multiple target genes, and on the other hand, it may act on pathways unrelated to ED or even opposite to the main target genes at the same time, which makes the final therapeutic effect of miRNA uncontrollable. Therefore, the mapping of miRNA target genes through rigorous, high-quality genomic and proteomic studies is a key step to achieving the clinical translation of miRNA. In addition, endothelial cells, smooth muscle cells, and immune cells constitute a complex microenvironment during the occurrence and development of ED, leading to the heterogeneity of miRNA expression. How to target miRNA to specific cells, effectively reduce the off-target rate, and minimize the toxic effects of miRNA on cells are also issues that need to be addressed. All in all, although results have been achieved at some magnitude and definite progress has been made in this field, several hurdles remain to be overcome before miRNAs can be formally used as therapeutic targets. Future studies should identify the target gene profiles of miRNAs and develop safe and efficient drug delivery platforms, to make miRNA therapy a clinical reality with bright prospects.

## Conclusion

In conclusion, the advances in the miRNA field so far continue to bring new surprises, but there is still a long way to go. ED remains a major problem affecting men’s health. Fortunately, new research on the pathogenesis of miRNAs in ED offers hope for miRNA-based therapeutic strategies for ED.

## Author contributions

Conceptualization: JS. Methodology: JS, KL. Investigation: JS, TS. Formal analysis: JS, KL. Visualization: WX, JW. Funding acquisition: JL. Supervision: JL. Writing – original draft: JS, JW. Writing – review & editing: JL. All authors have made substantial contributions to the study and have read and approved the final manuscript. All authors contributed to the article and approved the submitted version.

## Funding

This work was supported by the National Natural Science Foundation of China No.81873831.

## Conflict of interest

The authors declare that the research was conducted in the absence of any commercial or financial relationships that could be construed as a potential conflict of interest.

## Publisher’s note

All claims expressed in this article are solely those of the authors and do not necessarily represent those of their affiliated organizations, or those of the publisher, the editors and the reviewers. Any product that may be evaluated in this article, or claim that may be made by its manufacturer, is not guaranteed or endorsed by the publisher.
